# Temperate Bacteriophages—The Powerful Indirect Modulators of Eukaryotic Cells and Immune Functions

**DOI:** 10.3390/v13061013

**Published:** 2021-05-28

**Authors:** Martyna Cieślik, Natalia Bagińska, Ewa Jończyk-Matysiak, Alicja Węgrzyn, Grzegorz Węgrzyn, Andrzej Górski

**Affiliations:** 1 Bacteriophage Laboratory, Hirszfeld Institute of Immunology and Experimental Therapy, Polish Academy of Sciences, 53-114 Wrocław, Poland; martyna.cieslik@hirszfeld.pl (M.C.); natalia.baginska@hirszfeld.pl (N.B.); ewa.jonczyk-matysiak@hirszfeld.pl (E.J.-M.); 2 Laboratory of Phage Therapy, Institute of Biochemistry and Biophysics, Polish Academy of Sciences, Kładki 24, 80-822 Gdańsk, Poland; alicja.wegrzyn@biol.ug.edu.pl; 3 Department of Molecular Biology, University of Gdańsk, Wita Stwosza 59, 80-308 Gdańsk, Poland; grzegorz.wegrzyn@biol.ug.edu.pl; 4 Phage Therapy Unit, Hirszfeld Institute of Immunology and Experimental Therapy, Polish Academy of Sciences, 53-114 Wrocław, Poland; 5 Infant Jesus Hospital, The Medical University of Warsaw, 02-006 Warsaw, Poland

**Keywords:** prophage, immune system, lysogen, immunomodulation

## Abstract

Bacteriophages are natural biological entities that limit the growth and amplification of bacteria. They are important stimulators of evolutionary variability in bacteria, and currently are considered a weapon against antibiotic resistance of bacteria. Nevertheless, apart from their antibacterial activity, phages may act as modulators of mammalian immune responses. In this paper, we focus on temperate phages able to execute the lysogenic development, which may shape animal or human immune response by influencing various processes, including phagocytosis of bacterial invaders and immune modulation of mammalian host cells.

## 1. Introduction

Bacteriophages are viruses which infect bacterial cells. As bacterial parasites, they use host cells (bacteria) for amplification and to spread in the environment. Phages specifically infect bacteria using two alternative life cycles: lytic or lysogenic. Lytic (virulent) phages, after adsorption on a bacterial cell, inject their nucleic acid (DNA or RNA) inside it, while the capsids remain outside the cell. Bacterial DNA is degraded while the phage nucleic acid replication and phage protein synthesis occur the cell. In the next stages, progeny virions are assembled, and at the end of the cycle, they are released from bacterial cells, causing its disintegration and limiting bacterial population. In the lysogenic cycle (temperate phages), the predominant mechanism is the integration of the phage nucleic acid into the host genome, which does not result directly in the destruction of bacteria. Other temperate bacteriophages, such as *Escherichia coli*-specific P1 phage, can be localized extrachromosomally in the bacterial cell. Temperate phages have a significant impact on individual bacterial cells, as well as bacterial clusters. Their genes may replicate during bacterial genome replication without the assembly and release of new virion particles as well as with no lysis of the bacterial host. The integrated or extrachromosomal form of phages is called a prophage. A bacterial cell with a prophage integrated into its genome is called a lysogen [1,2,3]. Lysogenic development is characteristic for phages forming episomes or those integrating their genomes with the bacterial chromosome. External stressors (especially those causing a threat to the bacterial host cell and causing the induction of a stress response, like the SOS response) may initiate switching from the lysogenic to lytic cycle. An unusual phenomenon is communication between SPbeta bacteriophages via specific peptides in order to decide which cycle to enter [4]. In the described mechanism, in a lytic phage that encounters a bacterium (in this case *Bacillus subtilis*) for the first time, genes responsible for the inhibition of the lysogenization process (the *aimX* gene and the activator gene, *aimR*) are expressed. The lytic process continues in which the phage amplifies and destroys the bacterial host. At the same time, the AimP protein, encoded by the *aimP* gene, is secreted into the culture medium and processed into a mature peptide, and its appropriate concentration influences the switching of the phage to the lysogenic cycle. The lytic development is profitable for temperate bacteriophages when number of phages infecting a single bacterial cell is low, and there are many cells around which progeny phages can infect again after their liberation from the primary host. Under such conditions, production of numerous bacteriophages from a single predecessor is likely. On the other hand, when there are many phages around, lysogenization appears to be a more profitable strategy, as it is unlikely that progeny phages produced in an infected cell under such conditions might find next hosts for efficient propagation. Therefore, development of the above-described communication system, called the “arbitrium” system, allows phages to sense their density relative to host cells, making the choice between lytic and lysogenic development precisely controlled.

Surprisingly, metabolites of one bacterial species (in this case *Pseudomonas*) may induce a prophage internalized with the genome of other bacterial strain (switching the cycle from lysogenic to lytic phages associated with *Staphylococcus aureus*) [5].

*Escherichia coli* viruses Mu and λ, which are well-studied temperate phages, can integrate their genomes into DNA of bacteria via random transposition (Mu phage) [6] or site-specific recombination (λ phage) [7]. Genomes of other temperate *E. coli* bacteriophages do not integrate into bacterial chromosomes, but they exist as either linear (N15 phage) [8] or circular (P1 phage) DNA molecules [9]. Moreover, some temperate bacteriophages, like the P4 phage, require the complicity of another phage for their lytic propagation—in the case of P4, the helper role is played by the P2 phage [10,11,12].

A large body of data on the influence of lytic bacteriophages on the mammalian immune system is already available [13]. Lytic phages are of great importance, especially in phage therapy, due to the direct destruction of specific pathogenic bacterial cells [14,15,16,17,18]. In vivo studies using a mouse model of urinary tract infection showed that the intraperitoneal administration of bacteriophages improved the bactericidal activity of splenic phagocytic cells [19]. There are also studies in which it has been reported that the use of therapeutic phage preparations, including those against *E. coli* or *S. aureus* infections, did not downregulate the action of peripheral phagocytes, both in vitro [20] and in patients undergoing phage therapy [21]. Another well-described interaction of phages with mammalian cells is the inhibition of the formation of LPS-dependent reactive oxygen species (after T4 phage administration) by polymorphonuclear lymphocytes exposed to LPS (lipopolysaccharide) [22]. There have been reports in the literature on the influence of bacteriophages reducing the levels of pro-inflammatory cytokines on murine models of acute pneumonia [23] and wound infection [24] caused by *Acinetobacter baumannii*. On the other hand, the treatment of various immunocompetent cells, including mouse blood and bone marrow dendritic cells, with T4 phages or their proteins, did not cause significant changes in cytokine and reactive oxygen species concentrations [25]. The presence of strongly immunogenic components in phage virions, exemplified by T4 phage head proteins gp23* and Hoc, leads to the production of phage inactivating antibodies. This must be taken into consideration to assess the possible impact of phages on the human immune system [26,27]. It turns out that temperate bacteriophages can also significantly affect the human immune system, modulate it, and enhance or weaken the response of immunocompetent cells to infections.

**Figure 1:**
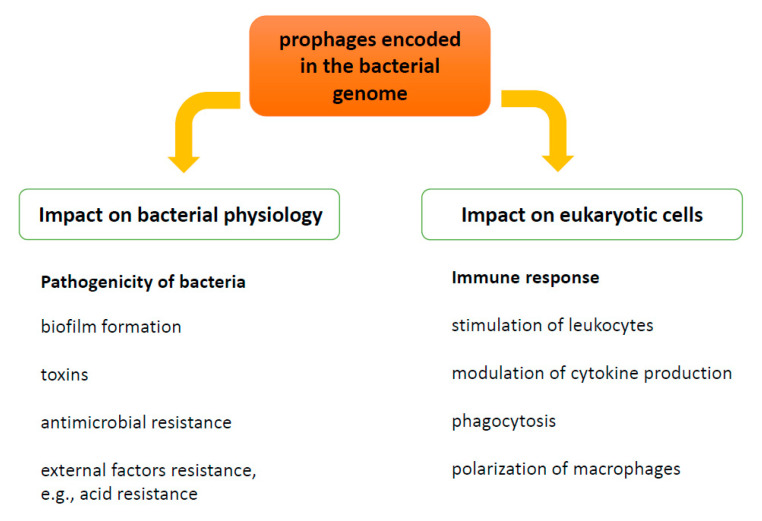
Prophage present in the bacterial genome may influence both pathogenicity of bacteria and modulate the functions of cells of immune system.

In light of this, the aim of this review article is to emphasize the impact of temperate bacteriophages on bacterial physiology and virulence, as well as on the animal and human immune system response in the process of destroying bacterial pathogens, which modulates the immune response [28,29] as well as susceptibility of the host organism to infections [30] (Figure 1). Currently, the association of phages with human health has been discussed and both beneficial and harmful effects have been considered [31]; therefore, this review will make additional arguments for this discussion.

### 1.1. Prophages as a Modulators of Bacterial Pathogenicity—Benefits for Bacteria

Phages are important vectors in horizontal gene transfer among bacteria. This process is related to interactions of bacteriophage genetic material with the bacterial genome and disruption of bacterial genes [32]. Bacteriophages are able to participate in gene transfer between bacteria in two ways: lysogenic conversion and transduction. In lysogenic conversion, temperate bacteriophages transfer advantageous genes to bacterial cells. As shown by Bossi et al. (2003), lysogenic strains of *Salmonella enterica* released prophages at low titers. These phages caused an efficient reduction of nonlysogenic-competing bacterial strains. On the other hand, some of the bacterial cells that acquired the prophage became resistant to killing and began to multiply, which may indicate the transmission of genes beneficial for bacteria by the mentioned prophage [33]. It has been proved in other studies that prophage (Mu, λ or P2) formation in *E. coli* cells has a beneficial impact on growth of bacteria compared to non-lysogenic strains [34,35,36]. The above-mentioned cases showed that prophages may contribute to a higher level of bacterial survival, and they can protect lysogenic cells against lytic infection of other phages [33].

It is postulated that transduction is a mutualistic process involving cooperation between prophages and their bacterial hosts [37]. Following bacteriophage infection of a bacterial cell, the transduced fragments of bacterial DNA can be packaged into forming bacteriophage heads instead of viral DNA [38,39,40]. Thus, phage particles are able to transfer genes from one bacterial cell to another. Bacteriophages mediate horizontal nucleic acid transfer in many species, including obligatory or facultative pathogens, like *E. coli, Streptomyces* spp. [38], *Salmonella* spp. [39], or *Listeria* spp. [40]. Bacterial viruses may deliver genes encoding virulence factors, such as enzymes, invasion effector proteins, proteins altering antigenicity, extracellular toxins, and other proteins to bacteria which increase their pathogenicity [41].

Virulence factors encoded by bacteriophages can be effectively produced and secreted by bacterial machinery during the lysogenic cycle. This type of secretion has been described for the cholera toxin. In contrast, the Shiga toxin is released from bacteria after its lysis. Lysogenic conversion to the lytic cycle occurs in altruistic bacterial populations, where some bacteria are sacrificed for the benefit of the entire population [42].

#### 1.1.1. Toxin Production

Since bacteriophage λ was considered for a long time to be the common model of a temperate phage, our knowledge on lysogenic cells came mostly from studies on this phage [43,44,45]. Because the vast majority of λ prophage genes are kept silent, with the exception of the *c*I gene coding for λ repressor which blocks expression of other phage genes while stimulating its own transcription, it was believed for years that prophages are generally inactive genetic elements [44]. Therefore, discoveries that other prophages might have a significant impact on the host’s physiology were quite surprising. In fact, the effects of prophages were considered previously in the light of their induction and expression of genes during subsequent lytic development. In particular, this knowledge concerned genes coding for toxins which make host bacteria pathogenic. When unicellular eukaryotic predators, like some ciliates (e.g., *Tetrahymena*) hunt for bacteria, they excrete hydrogen peroxide to weaken their prey. This strategy works well in most cases, however, when *E. coli* cells are lysogenic for Shiga toxin-converting bacteriophages, hydrogen peroxide causes prophage induction in a small part (up to 1%) of the bacterial population. This is enough to produce a large amount of Shiga toxins in the cells and cause their release after phage-mediated cell lysis. The toxins are able to kill the predator; thus, the rest of bacterial population (99% of cells or so) can survive. This strategy has been called “bacterial altruism” and exemplifies another way in which the presence of the prophage may influence survival of the host, this time when considering the whole population of lysogenic bacteria rather than a single cell [46]. Bloch et al. (2018) investigated 46 chemical compounds and their impact on *E. coli* prophages encoding Shiga toxin [47]. Three compounds (CM032D, CM092 and CM3186B) prevented the expression of prophage genes. Bacterial cells were treated with H_2_O_2_ to induce oxidative stress. The addition of the three mentioned compounds caused inhibition of expression of oxidative stress genes in *E. coli*. Reducing the effect of the stress factor contributed to the prevention of the release of prophage-encoded Shiga toxin in *E. coli* and the prevention of bacterial pathogenicity [47].

Examples of other bacterial toxins encoded by bacteriophage genes are botulinum neurotoxin produced by *Clostridium botulinum*, the TncA toxin produced by *C. novyi,* TcdA and TcdB toxins produced by *Clostridioides difficile*, the cholera toxin produced by *Vibrio cholerae*, enterotoxin A produced by *Pseudomonas aeruginosa*, and the diphtheria toxin produced by *Corynebacterium diphtheriae.* Since bacterial virulence, which is dependent on phage-encoded toxins, has been excellently reviewed recently [48,49,50,51], this phenomenon will not be discussed in detail here. Nevertheless, it is worth mentioning that new toxins encoded by bacteriophages are still being discovered, which can be exemplified by a binary toxin that is encoded by *cdtR*, *cdtA,* and *cdtB* genes of the phiSemix9P1 prophage of *C. difficile* [52].

Another group of bacterial toxins that are encoded by temperate phages are polymorphic toxins, which include the MuF family of toxins. These toxins are responsible for killing competing bacteria or inhibiting their growth [53,54]. The gene coding for MuF toxins is present in genomes of prophages found in chromosomes of various bacteria belonging to *Firmicutes* [55]. Thus, MuF toxins represent phage-encoded proteins that are used by the host to compete with other bacteria present in the environment.

Expression of gene encoding toxins depends not only on the genes of the prophages [56]. Interesting toxin gene (such as *speA*, *speB*, *speF*, *speG* or *smeZ*—genes encoding superantigens) expression regulation was described for bacteria incubated together with pharyngeal cells, and in the model of mouse infection [56,57]. As described for *Streptococcus pyogenes*, a non-toxigenic strain was converted to toxigenic bacteria by the scarlet fever filtrate agent [41]. A mammalian host may have an indirect or direct impact on gene expression of prophages in pathogenic strains [41,42]. In fact, *S. pyogenes* hyaluronidase is a phage-encoded protein, as are pyrogenic exotoxins. Moreover, production of the antiphagocytic M protein is stimulated in the presence of SP24 or SP44 prophage [42].

As demonstrated for the MGAS315 *S. pyogenes* strain (serotype M3), treatment with mitomycin C promoted induction of prophages present in this bacterial strain. It led to an increase in the number of virulence gene copies in prophages. However, mitomycin C treatment caused a decrease in the abundance of virulence factors interacting with the immune system. In addition, virulence particles may associate to phages after their induction. Proteins encoded by phages (superantigens and DNase enzymes) may presumably bind to proteins on the phage tail as was shown for hyaluronidase in *Streptococcus* A [58], which may lead to a reduction in the number of virulence factors in supernatants. Phage Φ315.4, encoding exotoxin K (SpeK), was induced during incubation with human epithelial pharyngeal cells (D562), but supernatants did not contain the toxic protein. However, transcription of the gene coding for streptococcal pyrogenic exotoxin A (SpeA) increased in human cells after incubation with *Streptococcus* A [59].

#### 1.1.2. Biofilm Formation

Biofilm formation has a great impact on bacterial physiology and survival, allowing cells to resist various deleterious environmental factors [60]. Biofilm is of great importance in the pathogenicity of many bacteria, including *P. aeruginosa*, making it extremely difficult to fight these bacteria [61]. The role of bacterial biofilm is particularly marked in the development of diseases such as periodontitis and caries [62,63], pneumoniae [64], or urinary tract infections [65]. Surprisingly, it was found that prophages have an important role in production of biofilms by various bacterial species. *Bacillus anthracis* is not able to form biofilm when deprived of prophages. Prophage Wip4 encodes an RNA polymerase sigma factor, which is responsible for activation of expression of genes, the products of which are necessary to form the biofilm [66]. In *P. aeruginosa* cells included in the biofilm, the most efficiently expressed genes were determined to be those present in the genome of the Pf4 prophage [67]. Extracellular DNA is a crucial element of a regular biofilm produced by various bacteria. It was demonstrated that such DNA appears in the biofilm formed by *Streptococcus pneumoniae* due to spontaneous induction of the SV1 prophage and lysis of a small fraction of cells initially included in the immature biofilm structure [68]. This might be considered as another example of ‘bacterial altruism’, when a small fraction of bacterial cells is sacrificed to facilitate survival of the rest of the population.

#### 1.1.3. Antimicrobial Resistance

Bacterial cells with prophages incorporated with their genomes may be more resistant to antimicrobial agents [69]. Studies have been conducted to search for the causes of drug resistance of *S. pyogenes* strains obtained from pediatric patients suffering from acute infections [70]. After the use of mitomycin C, it appeared that the prophages embedded in the bacterial genomes were responsible for inducing resistance to one or more antibiotics (such as clindamycin, lincomycin, chloramphenicol, or various macrolide antibiotics). In addition, in epidemic strains of methicillin-resistant *Staphylococcus aureus* (MRSA), the process of transduction of plasmids carrying penicillinase genes *blaZ* (decomposing natural penicillins) as well as genes of tetracycline resistance has been described [71]. Furthermore, in the genome of *A. baumannii,* many different genes encoding proteins related to the antimicrobial resistance, such as NDM-1 or OXA-23, were found [72]. Interspecies transduction of antimicrobial resistance genes has been proven for *Enterococcus* strains (for example, *E. gallinarum*, *E. faecalis*, *E. faecium*) collected from pigs [73]. Interestingly, lysogenic *E. coli* cells revealed increased tolerance to two antimicrobial compounds: chloroxylenol and 8-hydroxyquinoline [69]. 

#### 1.1.4. Bacterial Host Physiology

Recent studies indicate that the presence of prophages can significantly change the host cell physiology even at the lysogenic state, without the prophage induction. When proteomic and transcriptomic studies were performed with the *Escherichia coli* strain sensitive to the Shiga toxin-encoding lysogenic bacteriophage Φ24_B_, expression of 26 phage genes was detected, among which three were evidently expressed in the lysogenic state (not after spontaneous prophage induction) [74]. Physiological analyses indicated that *E. coli* lysogenic with Φ24_B_, were more resistant to acid (incubation at pH = 2.5) than non-lysogenic cells [75]. Production of the CII protein, encoded by the phage, appeared responsible for this acid tolerance. In fact, expression of the *gadABC* operon, which is involved in controlling the acid resistance mechanism, as well as *gadE, gadX,* and *gadW* regulatory genes, was evidently increased in lysogenic cells relative to controls [75]. In contrast to phage Φ24_B_, in bacteriophage λ, the *c*II gene cannot be expressed from the prophage. Moreover, other prophages could upregulate production of proteins of the type III secretion system in enterohemorrhagic *E. coli* strains [76]. Therefore, the presence of certain prophages in genomes of *E. coli* can significantly change physiological properties of the host. This statement was confirmed by even more spectacular results, indicating increased rates of respiration and cell proliferation of strains lysogenic for Φ24_B_, compared to non-lysogenic strains. Enhanced production of biotin and fatty acids was the cause of this phenomenon [69]. This appears to be an important feature which could increase bacterial survival under certain growth conditions.

As was shown, temperate phages modify bacterial host biochemical pathways and affect communication between infected and non-infected cells. Engineering of the bacterial host genome by prophages has a beneficial impact on bacterial cell processes [77]. Prophages affect the bacterial host metabolism as they modify bacterial resistance to other bacteriophages, development of bacterial cells, or production of virulence factors [78]. After release, these bacteriophages are able to lyse competitor strains and genetically and physiologically alter communication between bacteria [79]. Moreover, a phage-mediated release of intracellular substances, like nutrients for surrounding cells, was described [80]. Undoubtedly, phages can cause lysis of other bacteria while being harmless to cells lysogenic for the same kind of phage [81]. Finally, temperate bacteriophages also play an important role in protection of their host against various phage infections [2].

### 1.2. Prophages as Modulators of Gut Microbiota

It has been known for a long time that microorganisms which naturally inhabit the digestive system are extremely important in maintaining proper homeostasis, and thus the suitable functioning of the immune system [82]. The variety of human microbiota can be controlled by phages present in the intestinal tract. Based on the modern techniques of viral genome sequencing, it is argued that there is great variability in the virome among individuals, and only a small fraction of intestinal phages are common [83]. Reyes et al. (2010) hypothesized that temperate bacteriophages represent the majority among intestine viruses [84]. As demonstrated, 10^9^–10^10^ phage particles and 10^11^–10^12^ bacterial cells may be present in 1 g of dry weight of human feces. However, other results suggested that the concentration of phages may be higher, 10^10^–10^12^ bacteriophage particles per gram of stool [85,86]. There are data estimating that about 82% of adult gut bacteria are lysogens which contain the genetic material of prophages in their genomes [87]. Attention is also paid to the possibility of induction of prophages from intestinal bacteria under the influence of commonly used drugs [88].

Bacteriophages of bifidobacteria affect the diversity and composition of intestinal strains. Maternal bifidophages are able to transfer to infants and regulate diversity of infant bifidobacteria and composition in the infant gut [89,90]. It was found that adhesion of pathogenic bacterial strains to human HT29 cells was reduced after administration of the probiotic, lysogenic strain of *S. thermophilus* J34. This suggests that probiotic strains with prophages may have beneficial effects in the human gastrointestinal tract [91]. It is estimated that over 3% of the bifidobacterial genome consists of bifidoprophages [92,93,94]. This may be beneficial for bacterial strains, but on the other hand it may also cause host cell lysis [94]. Immediately after birth, the diversity of infant phages remains at a high level, while one week after delivery this correlation has a decreasing tendency [95,96]. It was suggested that transmission of maternal bacteriophages occurs through the activation of microbial prophages in milk, vagina, or placenta tissue [87,95,97]. Microscopic analyses of faecal and caecal samples showed that, in the adult human gut, most of the detected phages were derivatives of activated prophages from *Podoviridae, Siphoviridae,* and *Myoviridae* families [86]. Phages may alter the microbiological composition in the infant gut by reduction of dominant bacteria, and they allow other strains to develop, creating a variety of microbial community. Thus, phages play an important role in the evolution, diversity, and composition of bifidobacteria in the human gut [90].

The ability of adhesion to human intestinal epithelial cells (HT29), and impact on inhibition of the adhesion of pathogenic bacterial strains, were examined for two yoghurt probiotic strains of *Streptococcus thermophilus*. The adhesion of *S. thermophilus* J34 lysogenic strain to HT29 cells was considerably higher (34%) than that observed for the J34-6 strain without prophage (26%). The authors investigated the impact of *S. thermophilus* on the adhesion of three pathogens (*Listeria monocytogenes* Scott A, *Staphylococcus aureus* 6732, and *Salmonella enteritidis* S489). Competition, exclusion, and displacement assays were determined for both J34 and J34-6 probiotic strains. Bacteria were incubated simultaneously with enterocytes and three pathogenic strains (competition assay), or *S. thermophilus* cells were incubated with enterocytes, and after 30 min of incubation pathogens were added (exclusion assay), or enterocytes were incubated with pathogens, and after 30 min of incubation, *S. thermophilus* cells were added (displacement assay). The highest inhibition rate was observed for the J34 strain, which reached 63% for inhibition of *L. monocytogenes* by *S. thermophilus* J34 in the exclusion assay, 85% for *S. aureus* in the displacement assay, and 53% for *S. enteritidis* in the displacement assay. However, *S. thermophilus* J34-6 did not inhibit adhesion of *S. enteritidis* to human cells. Adhesion of *S. aureus* was the most efficiently inhibited in the exclusion assay (50%), whereas *L. monocytogenes* was inhibited at the level of 42%. These results showed that probiotic strains can play an important role in the protection of the human gastrointestinal tract against invasion of pathogenic bacteria [91].

The presence of prophage genes in the genome of *Faecalibacterium prausnitzii* strains, which are considered to be one of the most important components of human intestinal microbiota, was also described. It is hypothesized that there may be a positive correlation between the presence of temperate phages and the development of intestinal inflammation (in particular IBD—inflammatory bowel disease) associated with a reduced amount of these bacteria [98]. It is also presumed that the presence of prophage-encoded genes (such as genes for the components of the Panton-Valentine leucocidin (PVL) in MRSA strains) may increase the ability of intestinal bacteria to damage the intestinal wall and inhibit phagocytes [99].

## 2. Influence of Prophages on Human Immune Response

### 2.1. Phagocytosis and Intracellular Killing of Bacteria

Phagocytosis, one of the types of endocytosis, is an extremely important mechanism of innate immunity, helping to fight various pathogens. The most emphatic phagocytic cells in the human immune system are monocytes/macrophages and neutrophils, but dendritic cells, eosinophils, and basophils also have the ability to intracellularly kill pathogenic microorganisms. The role of this process is emphasized mainly in combating bacterial infections [100]. The whole arsenal of cytotoxic agents directed against bacteria (including both oxygen-dependent and independent mechanisms), after their absorption by phagocytes, causes a series of occurrences leading to its elimination [101]. Some drug molecules can be absorbed by cells by phagocytosis, and can also inhibit this process [102], so it is important to understand the influence of various factors on endocytic processes. Due to the fact that many bacteria have integrated prophages in their genomes, which can reveal their presence only under certain conditions, at some point, attention began to be paid to the possible influence of these temperate phages on the phagocytes and the process of intracellular killing.

It has been found that prophages can modulate the human immune response to bacterial infection [30]. Research conducted by Młynarczyk et al. (1989) described a lysogenic conversion process which may influence the susceptibility of a *Staphylococcus aureus* strain to phagocytosis [103]. The authors examined intracellular killing of non-lysogenic *S. aureus* strain 8325-4 and its eight lysogenic variants by granulocytes isolated from human blood. After 60 min incubation, the level of intracellular killing was assessed. For lysogenic strains, the level of intracellular killing after one hour oscillated between 29–38%, while for the non-lysogenic strain it was 63%. These observations proved that the lysogenic staphylococcal 8325-4 strain was less susceptible to intracellular killing by granulocytes compared to the strain without the prophage. It is probable that the observed phenomenon is related to the prophage genes, which may affect the synthesis of the antiphagocytic surface receptors or may result from the presence of R plasmids in cells. The authors also examined the influence of prophage presence in *S. aureus* 8325-4 on the intensity of leukocyte stimulation using a bioluminescence test. The results showed that bioluminescence of leukocytes after lysogenic strain stimulation was lower than that observed with non-lysogenic strains.

Furthermore, other studies investigated the effect of group F prophages on the bacterial stimulation of human leukocytes [104]. Chemiluminescence of leukocytes for the lysogenic *S. aureus* 8325-4 strain was at the level 15.4–37.2% compared to a control strain without the prophage (100% of bioluminescence). Intracellular killing of the non-lysogenic strain after 30 and 60 min was at the level 19 and 63%, respectively. For two strains with prophages, after 30 min of incubation with human leukocytes, the level of intracellular killing was very low, and after 60 min, 32–38% of bacteria were killed. The reduction of leukocytes stimulation by lysogenic strains can be associated with an increase in the pathogenicity of bacteria that have prophage genes integrated into their genome, compared to the strains without the prophage.

Interestingly, genes of the Pf prophage are very widespread in the genome of biofilm-forming *Pseudomonas* strains [67,105]. Furthermore, the growth of the *P. aeruginosa* biofilm promotes production of the Pf phage [106]. As demonstrated in a murine model of pneumonia, *P. aeruginosa* biofilm formation and Pf prophage production inhibit bacterial dissemination from lungs to other tissues. In addition, the mentioned phages contributed to the inhibition of *P. aeruginosa* invasion of airway epithelial cells. This suggests that Pf phage particles may interact with the bacterial host in lungs. In vivo studies showed that the Pf phages inhibit recruitment of neutrophils, reduce levels of cytokines, and protect lungs from damage caused by infection. Moreover, production of Pf phages by bacteria results in less efficient phagocytosis by macrophages in vivo, compared to the non-lysogenic strains [106].

The reduction of phagocytosis of pathogenic *Pseudomonas* strains due to the presence of temperate Pf bacteriophage has been known for a long time and widely described [107]. Pf phages internalized in the endosomes of phagocytic cells activate TLR3 receptors, which leads to the stimulation of extracellular secretion of interferons, which in turn inhibit TNF-α secretion. The silencing of pro-inflammatory mediators weakens the phagocytosis of pathogenic bacteria [107].

Secor et al. (2017) studied the effect of a *P. aeruginosa* strain lysogenic with the Pf4 phage, in comparison to a bacterial strain without a prophage, on the polarization of bone marrow-derived mouse macrophages [106]. It turned out that the prophage significantly promoted the polarization of macrophages towards the M2 type, a phenomenon which causes suppression of the inflammatory response, for example, by producing anti-inflammatory markers such as IL-10. As a consequence, it was observed that the bacterial strain producing the filamentous Pf4 phage was less susceptible to phagocytosis by macrophages than the strains without the Pf prophage.

Other studies described the FIZ15 bacteriophage, obtained from the clinical *P. aeruginosa* strain, which caused the lysogenic conversion in the *Pseudomonas* PAO1 strains [108]. Interestingly, this process contributed to the attenuation of phagocytosis of bacteria by murine macrophages. The presence of a prophage in the bacterial genome and the resulting change in its surface provoked an increase in resistance to neutralization by human serum (several or several dozen times higher survival rate of bacteria).

The design of vaccines against phage virions, which increase the pathogenicity of bacteria, offers an attractive idea in the prevention of severely treatable infections. Sweere et al. (2019) described a filamentous Pf phage from the *P. aeruginosa* strain, whose presence positively correlated with the development of chronic wound infection in mice and humans. It was associated with an increase in the production of TLR3-dependent interferon type I after phage endocytosis by leukocytes, a weakening of TNF-α secretion, and, consequently, inhibition of phagocytosis of bacteria. In contrast, mice immunized against pseudomonal bacteriophage were protected against infections caused by *P. aeruginosa* [109].

Prophage excision may cause stimulation of host gene expression [110]. Such a phenomenon was described for *Listeria monocytogenes* serovar 1/2, where excision of the prophage causes activation of the *comK* gene, otherwise disrupted by prophage integration. Since a *L. monocytogenes* Com system is necessary for bacterial virulence during intravenous infection of mice [111], reactivation of the *comK* gene allows for effective infection of mammalian cells. In addition, the expression of the *comK* gene is associated with a greater probability of survival of bacteria into the macrophage and with promoting the escape of bacteria from phagocytosis. This is a great example of the evolution of prophages to cooperate with their bacterial host in an intracellular environment [112].

### 2.2. Cytokine Production and Function of T-Cells

Cytokines—small, low-molecular-weight peptides—are of great importance in signaling and communication between cells of the immune system, along with the endocrine, paracrine, and autocrine pathways. Many immunocompetent cells produce and secrete cytokines (including T and B lymphocytes, macrophages, mast cells, fibroblasts, etc.) and in addition more than one type of cell may produce a particular cytokine [113]. They may act by up-regulating inflammatory processes (pro-inflammatory cytokines), or by stimulating the chemotaxis of immune cells, including phagocytes, to the site of inflammation (chemokines), or on the other hand, they may also be responsible for suppressing the inflammatory process and stimulating tissue repair [114]. It turns out that both lytic and temperate phages can have immunomodulatory properties, which can both facilitate the spread of bacteria and increase their pathogenicity by weakening the human immune response, as well as enhance the immune system, which, in two ways, can lead to the destruction of tissue and organs, or the effective elimination of the pathogen.

The lysogenic conversion region in *S. pyogenes* strains contains genes for streptococcal pyrogenic toxins. Various *S. pyogenes* strains express different types and numbers of toxic proteins [115]. The action of toxins leads to an abnormal activation of the immune system. These superantigens are able to incorrectly bind the major histocompatibility complex (MHC) proteins, especially the MHC class II β chain, as well as T-cell receptors (TCR) [116], which in turn is closely related to an increase in the levels of pro-inflammatory mediators such as IL-2, and cytokines secreted by stimulated T lymphocytes such as TNF-α of IL-1β, and this is inextricably linked with the spread of inflammation [117,118]. Some of these phage-associated antigens have been found to be mitogens for leukocytes isolated from human blood, inducing the process of lymphocyte mitosis and their increased proliferation [116].

It has been shown that temperate bacteriophage genes constitute an extremely important component of the genome of the *S. aureus* Newman strains [119]. Various strains of a given species of bacteria may vary greatly in their ability to induce acute adaptive immune responses in humans. Sela et al. (2018) have demonstrated that this is associated with dramatic differences in the intensity of the specific protective T helper responses (Th1 and Th17 lymphocytes). Importantly, those differences were observed using multiple human donors, which suggests an association with pathogens and not with an individual ability to mount an immune response. The deletion of prophages (ϕNM1-4) from the bacterial genome caused a marked reduction of T cell responses. That study indicated that the heterogeneity of the human immune response to various strains of bacteria depends largely on their prophage content, and thus, sheds more light on the well-known phenomenon of the variability in human susceptibility to bacterial infections [30].

Phages are able to conduct gene transfer between each other during polylysogenic state establishment, and are associated with immune system action [120]. Moreover, many bacterial strains replaced the *rfb* gene region [121] during horizontal gene transfer, which reduces the immunogenicity of bacteria. The *rfb* gene encodes proteins necessary for the synthesis of bacterial LPS. Since bacterial LPS is a highly immunogenic antigen [122,123], removing this gene results in weakening of stimulation of the immune system after entry of the bacteria into an organism. The phenomenon of replacement in the *rfb* locus by stx1 and stx2 bacteriophages has been described for *E. coli* strains [121,124].

Bae et al. (2006) reported the clinical isolate of *S. aureus* Newman, which contains in its DNA prophage sequences: ϕNM1, ϕNM2, ϕNM3, and ϕNM4 [28]. The authors suggested that specific genes of these four prophages are responsible for the pathogenicity of *S. aureus* strains. Moreover, studies demonstrated that ϕNM3 prophage carries genes *scn, chp, sea,* and *sak,* which modulate the human innate immune system and play a significant role in infections caused by staphylococci [125,126]. Expression of the above-mentioned genes affects processes related to the presentation of antigens or complements activation in humans. For example, Dohlsten et al. (1993) observed 1000-fold higher activation of T cells in humans than in the murine model [127] after *sea* gene expression. In addition, staphylococcal mutants without the ϕNM3 prophage replicated less efficiently in liver cells of infected mice than *S. aureus* with inserted ϕNM3. These results showed that the ϕNM3 prophage can carry various genes affecting the functions of the murine innate immune system [28].

Interestingly, van Wamel et al. (2006) used 5 classical laboratory and 85 clinical *S. aureus* strains to investigate the distribution of temperate βC-ϕs phages [29]. Their results indicated that βC-ϕs were present in 88.9% of the analyzed staphylococcal strains. The presence of various virulence factors encoded by them was also described. SAK (staphylokinase), CHIPS (chemotaxis inhibitory protein), and two superantigens SEA and SEP were found in 76.6, 56.6, 27.8, and 7.8% of strains, respectively. The above-mentioned modulators of the human innate immune system may be easily and effectively transferred by β-hemolysin converting phages (βC-ϕs) between staphylococcal strains [29]. CHIPS (encoded by *chp* gene) is a protein influencing a stronger inhibition of calcium mobilization inducted by the complement C5a protein [126]. SCIN (encoded by s*cn* gene) is associated with the inhibition of the process of hemolysis in humans, and it inhibits the convertase enzyme and negatively affects the C3b complement protein adsorption on the bacterial cell surface. This inhibition protects *S. aureus* from phagocytosis by human neutrophils [128]. The CHIPS protein is able to bind two chemokine receptors. It leads to attenuation of the response of the receptor for C5a and receptor of formylated peptide of human neutrophils. Consequently, activation in response to formylated peptides and C5a is inhibited, as well as chemotaxis of human neutrophils. CHIPS as a virulence factor of *S. aureus* strains protects bacteria from the innate immune system [126,129,130,131]. SAK affects the innate immune system in various ways [132], for example, by inhibiting opsonization [133]. Staphylococcal superantigens have the ability to directly bind to the MHC class II molecules, which may lead to the activation of monocytes and, consequently, their stimulation to increase the secretion chemokines and other pro-inflammatory cytokines [134].

The Panton-Valentine leukocidin is a factor that significantly increases the pathogenicity of MRSA strains. Depending on the concentration, PVL causes necrosis or apoptosis of human cells in vitro due to the formation of pores in the cells or their organelles, such as mitochondria [135]. About 30% of isolated strains are PVL-positive and it is related to the presence of the prophage genes in their genome [136]. Interestingly, it was shown that the distribution of individual phages carrying the PVL toxin gene depended on the geographic location from which the bacterial strain was obtained [136]. PVL induces a pro-inflammatory response by binding to the monocytes and macrophages, resulting in the release of caspase-1-dependent cytokines (IL-18 and IL-1β). The cytotoxic effects on neutrophils also causes the release of PAMP (pathogen associated molecular patterns) and DAMP (damage associated molecular patterns) particles from them, and consequently an increase in the level of pro-IL-1β in monocytes and macrophages [137]. However, there is also contradictory evidence that PVL, at an appropriate concentration, may be protective and increase the ability of the immune system to counteract *Staphylococcus* infections [138]. As can be seen, the genes carried by the prophages significantly modulate the immune response (Table 1).

## 3. Conclusions

Due to production of specific phage-encoded toxins, prophages can be responsible for virulence of bacterial cells. Prophages may also influence host physiology, by modulating its resistance to various environmental factors (like low pH), enhancing tolerance to antibiotics, or facilitating biofilm formation. Therefore, the presence of prophages can be beneficial for their hosts, which suggest that bacteriophages may be considered as important modulators of the host physiology, often increasing their survival rate under specific environmental conditions.

As presented, bacteriophages can also affect eukaryotic cells differentially, and this effect also applies to temperate phages. Some virulence regions carried by phages may protect bacteria against the human immune system. Prophages may protect bacteria against the influx of human neutrophils caused by chemotaxis, inhibit both complement action, and/or opsonization of pathogens. They can also affect macrophage phagocytosis in vivo. All examples of modulating properties by phages demonstrate that bacterial viruses have a huge impact not only on bacteria but also on more complex multicellular organisms, including the human immune system. The presented data indicate the importance of prophages on modulation of the human immune response but those studies are still at an early stage and require more data. It may be anticipated that those data could contribute to novel treatment abilities based on prophage targeting. Interestingly, in a recent excellent review on phage interactions with the immune system, the significance of prophages is only briefly mentioned [139].

It is recommended that prophage-free bacteria be used for the amplification of subsequently applied phages in the therapy to ascertain its safety and lack of side-effects. However, the selection of such non-lysogenic bacteria is difficult or impossible, as the phenomenon of lysogeny is very frequent. Therefore, removal of prophages from such strains of bacteria is a suggested strategy. Those phage curing methods have been previously described [14].

## Figures and Tables

**Table 1:** The influence of the prophage present in the bacterial genome on the immune system.

Modulation of	Direction of Change
T cell proliferation	↑/↓ [30]
B cell proliferation	↑ [30]
IgG synthesis	↑ [30]
IFN-γ or IFN type 1 synthesis	↑ [30,109]
ROS production by leukocytes	↓ [103,104]
levels of leukocytes in the infected tissue (based on the presence of CD45 molecule)	↓ [106]
intracellular killing of bacteria (phagocytosis)	↓ [103,104,109]
macrophage polarization	towards M2 type [106]
pro-inflammatory cytokines (e.g., TNF-α, IL-1β, IL-2) in vivo in the infected tissue and in vitro	↓ [106,109] /↑ [117,118]
anti-inflammatory cytokines (e.g., IL-10) in vivo in the infected tissue	↑ [106]

## Data Availability

Excluded.
